# Diagnostic and Prognostic Role of Blood and Cerebrospinal Fluid and Blood Neurofilaments in Amyotrophic Lateral Sclerosis: A Review of the Literature

**DOI:** 10.3390/ijms20174152

**Published:** 2019-08-25

**Authors:** Delia Gagliardi, Megi Meneri, Domenica Saccomanno, Nereo Bresolin, Giacomo Pietro Comi, Stefania Corti

**Affiliations:** 1 Dino Ferrari Centre, Neuroscience Section, Department of Pathophysiology and Transplantation (DEPT), University of Milan, 20122 Milan, Italy; 2 Foundation IRCCS Ca’ Granda Ospedale Maggiore Policlinico, Neurology Unit, 20122 Milan, Italy; 3 Foundation IRCCS Ca’ Granda Ospedale Maggiore Policlinico, Neurmuscular and Rare Diseases Unit, 20122 Milan, Italy

**Keywords:** amyotrophic lateral sclerosis, neurofilaments, p-NfH, NfL, cerebrospinal fluid, blood, biomarkers

## Abstract

Amyotrophic lateral sclerosis (ALS) is a fatal neurodegenerative disorder affecting upper and lower motor neurons (MNs) that still lacks an efficacious therapy. The failure of recent therapeutic trials in ALS, other than depending on the poor knowledge of pathogenic mechanisms responsible for MNs loss, is largely due to diagnostic delay and the lack of reliable biomarkers for diagnosis, prognosis and response to pharmacologic intervention. Neurofilaments (Nfs) are neuron-specific cytoskeletal proteins, whose levels increased in biological fluids proportionally to the degree of axonal damage, both in normal and in pathologic conditions, representing potential biomarkers in various neurological disorders, such as motor neuron disorder (MND). Growing evidence has shown that phosphorylated neurofilaments heavy chain (p-NfH) and neurofilaments light chain (NfL) are increased in blood and cerebrospinal fluid (CSF) of ALS patients compared to healthy and neurological controls and are found to correlate with disease progression. In this review, we reported the most relevant studies investigating the diagnostic and prognostic role of Nfs in ALS. Given their reliability and reproducibility, we consider Nfs as promising and useful biomarkers in diagnosis of MND, early patient identification for inclusion in clinical trials, prediction of disease progression, and response to pharmacological intervention, and we suggest the validation of their measurement in clinical activity.

## 1. Introduction

Amyotrophic lateral sclerosis (ALS) is a fatal neurodegenerative disorder affecting upper and lower motor neurons (MNs) that still lacks an efficacious therapy. In the past decades, several studies have been led to find a molecule able to halt disease progression, but they were unsuccessful. The incomplete knowledge of pathophysiological mechanisms underlying ALS and the lack of available and reliable biomarkers for diagnosis, disease progression, prognosis, and response to pharmacologic intervention has limited the clinical experience about motor neuron disorders (MND) and has probably contributed to the failure of recent therapeutic trials in ALS.

An ideal biomarker would have higher levels of expression in patients compared with controls, would maintain steady levels throughout the disease course, being susceptible to treatment effects and disease exacerbations, and would reflect the rate of neurological decline and the disease duration.

Evidence showing that neurofilaments (Nfs) are found at elevated concentrations in cerebrospinal fluid (CSF) and blood of patients affected by ALS, has prompted the scientific research to explore their role as diagnostic and prognostic biomarkers in ALS.

**Figure 1:**
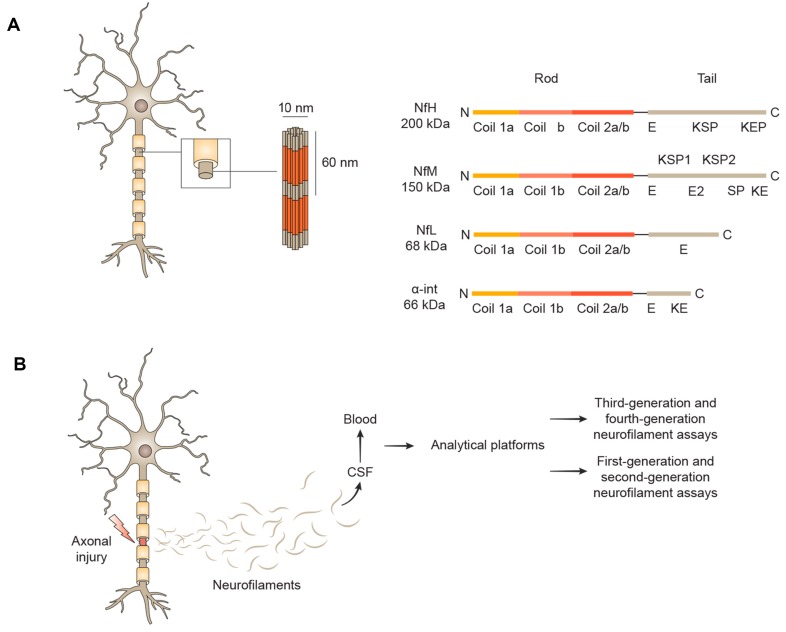
**A**. Overview of neurofilaments structure. Neurofilaments (Nfs) are cylindrical structure of 10 nm diameter abundantly expressed by large calibre myelinated axons. According to the molecular mass of their subunits, Nfs are classified in neurofilament light chain (NfL), neurofilament medium chain (NfM), neurofilament heavy chain (NfH) and α-internexin (α-int). Nfs subunits are formed by a conserved α-helical rod domain, a variable amino-terminal globular head region and a carboxy-terminal tail domain. Each carboxy-terminal tail is variably enriched of glutamic-acid (E), lysine-serine-proline (KSP), lysine-glutamic acid-proline (KEP), serine-proline (SP) and lysine-glutamic acid (KE) segments. **B**. Detection methods of Neurofilaments in biological fluids after axonal damage. Axonal injury induces Nfs release in extracellular fluids, and subsequently, given its proximity to central nervous system (CNS), into cerebrospinal fluid (CSF). First- and second- generation assays (immunoblot and enzyme-linked immunosorbent assay (ELISA), respectively) are able to measure Nfs levels in CSF, but present limited sensitivity for Nfs detection in blood. Third-generation (electrochemiluminescence) and fourth-generation assays (single-molecule array) can also detect ultralow concentrations of Nfs in blood, enabling longitudinal studies in patients and healthy controls (HC).

Nfs are neuron-specific cytoskeletal proteins with a characteristic diameter of 8–10 nm, and are members of the intermediate filament family (Figure 1A) [1]. They are present in cell bodies and axons and are involved in growth, stabilization and polarization of neural cells, enabling effective axonal conduction. According to the molecular mass of their subunits, Nfs are classified in light (NfL), medium (NfM) and heavy chain (NfH). Nfs subunits are formed by a conserved α-helical rod domain, a variable amino-terminal globular head region and a carboxy-terminal tail domain [2]. Assembly of neurofilament protein monomers in heterodimers, and then in tetramers, is followed by lateral association of eight tetramers, resulting in the formation of cylindrical structures, which in turns undergo an end-to-end annealing and a radial compaction to obtain the mature long neurofilament polymer with a diameter of 10 nm [3]. Post-translational modifications like phosphorylation and *O*-glycosylation are crucial for neurofilaments aggregation, especially in NfM and NfH [1].

Being highly expressed in large caliber myelinated axons, Nfs are released in the extracellular space and their levels increase in blood and CSF proportionally to the degree of axonal damage, both in normal and in pathologic conditions [4]. Physiological ageing has been associated with an increase of blood and CSF NfL levels, probably due to reduced CSF turnover, slowly progressive structural axonal damage, and metabolic alterations in Nfs turnover [2]. Moreover, a higher amount of Nfs has been found in patients affected by various neurological diseases, such as multiple sclerosis, where Nfs have been found to correlate with disability and disease activity, cerebrovascular diseases, traumatic brain injury and neurodegenerative disorders, among which Alzheimer’s disease, frontotemporal dementia, Parkinson’s disease and ALS [2]. In particular, it has been postulated that altered transport of Nfs down the axon and their subsequent accumulation in the cell body, proximal to the axonal process, may provoke MNs death in ALS, hypothesizing a role of Nfs in neurodegeneration.

Growing evidence showed that NfL and phosphorylated-NfH (p-NfH) are non-specific markers of axonal damage and are reported to be increased in CSF and blood of ALS patients, at higher levels compared to other neurodegenerative disorders or disease mimics [5].

In particular, CSF and serum p-NfH concentrations are significantly increased in ALS in comparison with controls and ALS mimics, representing a useful diagnostic biomarker, especially in early symptomatic phases of the disease. NfL elevation in biofluids is the first detectable event accompanying neurodegeneration in ALS, nevertheless they are less specific for MND, being increased also in other neurodegenerative disorders. Conversely, both plasma/serum NfL and p-NfH might be used as prognostic and pharmacodynamic biomarkers, since they are reported to correlate with disease severity and their blood levels remain stable over time.

In this review, we reported the most relevant studies investigating the role of Nfs in ALS. Describing the main findings of these works, we highlighted the advantages and the pitfalls of blood and CSF measurements of p-NfH and NfL, and we discuss their role as potential diagnostic and prognostic biomarkers in ALS.

Since their reliability and reproducibility, Nfs could represent diagnostic and prognostic biomarkers useful for precocious identification of patients and their inclusion in clinical trials, assessment of individual prognosis and response to experimental drugs.

## 2. CSF and Blood Neurofilaments as Biomarkers in ALS

The role of Nfs as diagnostic and prognostic biomarkers in MND has been suggested by several evidence showing that Nfs are found at elevated concentrations in CSF and blood of patients affected by ALS.

More than two decades ago, Nfs were discovered at higher levels (5 to 10 folds) in CSF of ALS patients, compared to healthy controls (HC) [6]. Given the proximity to the degenerating MNs in brain and spinal cord, CSF is expected to contain a greater concentration of Nfs (10 folds higher than blood) and could be a more suitable source for the study of these cytoskeletal proteins.

The improvement of new immunoassays able to detect proteins in biological fluids even if when present at low levels, has allowed Nfs measurement also in serum and plasma, providing the possibility to conduct longitudinal studies, useful to evaluate biological response to therapies.

Indeed, first- and second-generation Nfs assays, i.e. immunoblot and enzyme-linked immunosorbent assay (ELISA), respectively, were able to detect precisely only high concentrated CSF proteins, having a poor sensitivity in detecting Nfs in blood (Figure 1B). Conversely, third-generation assays (electrochemiluminescence), and especially fourth-generation assays (single-molecule array), can measure also ultralow concentrations of blood NfL, allowing to detect even subtle longitudinal changes in HC [2]. Further, blood samples are easily accessible and could be obtained in a lesser invasive way, providing the possibility to conduct longitudinal studies, useful to evaluate biological response to therapies.

Almost all the studies investigating both CSF and blood compartments on ALS patients have showed high correlation between CSF and blood Nfs concentrations, with increased Nfs levels than in blood [7,8,9,10,11,12,13].

Several studies have shown that both p-NfH and NfL are found at higher levels in CSF and blood of ALS patients, not only as compared to controls, but also to disease-mimics (DM), suggesting their potential utility in differential diagnosis. Furthermore, it has been investigated their role in distinguishing among different ALS phenotypes.

In a recent metanalysis including 15 studies, levels of CSF and blood Nfs in ALS patients were compared to HC, ALS mimic and neurological controls with CNS parenchymal involvement [14]. The main findings of this systematic review were that Nfs have actually a role in neurodegeneration in ALS, in particular, CSF p-NfH and NfL accurately discriminate between ALS and HC, and blood and CSF NfL could distinguish ALS patients from other neurological diseases. Nevertheless, Nfs are not specific for this disorder, so they could be likely used as marker of disease progression [14].

## 3. Phosphorylated Neurofilament Heavy Chain (p-NfH)

NfH undergo post-translational phosphorylation, which is relevant for Nfs transport along the axons and regulates axon stability and protein-protein interactions.

Compared to NfL, p-NfH are more stable and less susceptible to proteases degradation, representing a steady and reproducible biomarker in consecutive measurements. Nevertheless, the presence of immune response against NfH and the hook effect due to p-NfH aggregation in plasma samples represent an issue in accurate Nfs measurement, since they may accelerate p-NfH clearance or reduce the binding sites available for antibody binding through ELISA techniques [15].

Different studies have investigated the utility of p-NfH in blood and CSF samples of ALS patients (Table 1).

Boylan et al. compared p-NfH levels in plasma, serum and CSF in ALS patients, showing a significant association between higher concentrations and faster disease progression, evaluated as faster decline in ALS Functioning Rating Scale Revised (ALSFRS-R) [16]. Although to a lesser extent for CSF, increased serum and plasma p-NfH were associated with shorter survival in ALS patients. Moreover, they found a mild but significant increase in plasma p-NfH in patients with bulbar versus spinal onset. These findings support the hypothesis that blood and CSF p-NfH provide a reliable indicator of disease activity and prognosis in ALS.

Several studies did not find an association between Nfs concentrations and age at onset of ALS, suggesting a stronger relationship with clinical course than with age of the patients [16]. McCombe at al. found a significant association between p-NfH peak and age, but no relationship was found between age and the rise of p-NfH protein [17]. Nevertheless, age could be used as potential covariate in these studies.

Lu and colleagues lead a prospective study on 74 ALS affected patients, whose blood samples have been collected every three months for a period of three years [15]. Although they did not find a significant difference in plasma p-NfH at baseline between ALS and controls, they observed higher blood concentrations in patients with shorter disease duration and diagnostic latency, as if plasma p-NfH levels could reflect the speed of clinical decline and disease progression. Differently from results obtained in mouse models, in humans plasma concentrations of p-NfH would peak early in patients with a rapid and aggressive clinical course, decreasing later in the disease, while they would slowly increase from a low initial level in patients with slower disease progression. These results suggest a limited role of cross-sectional studies, since they would capture a random snapshot of a biological indicator, regardless of disease stage and clinical heterogeneity. Therefore, they discuss the prognostic role of plasma p-NfH and their utility in evaluating treatment response, since changes in plasma p-NfH levels in a follow-up period do not predict disease progression.

Similar results were obtained from McCombe 2015 et al., who found a significant difference in blood p-NfH levels at baseline between ALS and HC [17]. Despite a considerable variability, they showed an increasing trend of plasma p-NfH over time, which was inversely correlated with survival length: patients with rapidly progressive disease and shorter survival present higher p-NfH levels early, followed by a fall later in the disease, while lower levels have been observed in slower progressors. So, higher concentrations and rapid rise in p-NfH levels at the beginning of the disease, may predict disease severity and prognosis. They did not find a significant relationship between p-NfH levels and clinical variables like gender, upper motor neuron (UMN)/ lower motor neuron (LMN) predominant phenotypes, site of onset, use of riluzole and disability measured with ALSFRS-R.

Li and colleagues confirmed the presence of higher p-NfH levels both in plasma and in CSF of ALS patients in comparison to controls and they identified a cut-off level of 685 pg/ml for p-NfH in plasma (sensitivity of 80.39% and specificity of 73.81%), and the optimal cut-off value in CSF of 589 pg/mL, with a sensitivity of 82.35% and a specificity of 73.81% [7]. CSF p-NfH levels, more than blood p-NfH levels, showed an inverse correlation with time to generalization (TTG), and along with bulbar onset, seem to predict a shorter TTG in ALS patients.

In the retrospective study by De Schaepdryver et al. [8] CSF and matched serum concentrations of p-NfH were found to correlate well in ALS patients, with higher concentration in CSF, and were significantly increased in ALS versus disease controls (DC) and DM. To discriminate ALS from mimics, the authors identified a cut-off value of 750 pg/mL in CSF, with an elevated sensitivity and specificity (respectively 92.9% and 96%). For serum, the optimal cut-off distinguishing between ALS and DM was 81.9 pg/mL, with a lower sensitivity and specificity. Even if both serum and CSF p-NfH levels correlated with disease progression, only the latter was associated with the burden of UMN and LMN involvement, assessed by clinical and neurophysiological examinations. These findings have been confirmed by Poesen et al., who found a positive correlation between CSF Nfs and the number of regions with both UMN and LMN involvement [18]. Hence, the diagnostic potential of p-NfH measurement in CSF, more than serum, could be used as a criterion of inclusion in clinical trials.

The most relevant finding of the study lead by Zucchi et al. was that CSF and serum p-NfH can discriminate ALS with predominant UMN signs from other UMN syndromes, like hereditary spastic paraplegia (hSP) and primary lateral sclerosis (PLS); moreover, CSF p-NfH concentration may differentiate UMN syndromes from HC and are the most stronger predictors of survivals [9].

Finally, p-NfH in CSF have shown to be a good prognostic biomarker not only in sporadic ALS, but also in ALS affected patients harboring mutation in the C9Orf72 gene. Gendron and colleagues identified a cut-off value of 176 pg/mL for p-NfH in CSF, able to discriminate with elevated sensitivity and specificity (98.8% and 96.4% respectively) between symptomatic and asymptomatic C9Orf72 mutation carriers [19]. Moreover, higher p-NfH levels were associated with faster disease progression and shorter survival both into this subset of patients and when comparing C9-ALS patients with asymptomatic carriers and with non-C9Orf72 mutation carriers, likely reflecting an increased neuronal injury in C9-ALS patients. In longitudinal evaluation, CSF p-NfH levels remained stable over time. Similarly, Weydt and colleagues demonstrated that ALS symptomatic carriers presented 10-fold increased levels of p-NfH in comparison to asymptomatic carriers and HC [13]. According to their work, Nfs are normal in the asymptomatic phase onset and increased after onset of symptoms in ALS, representing a marker of neurodegeneration.

In a large prospective study on patients affected by MND, CSF p-NfH were increased as compared to MND mimics and HC, showing a cut-off level of 560 pg/mL, and were associated with progression and disease duration [20].

In the work of Poesen et al., CSF p-NfH were shown to have a diagnostic role, since they were able to discriminate with high sensitivity and specificity (90.7% and 88%, respectively) ALS patients from DM, with a cut-off of 768 pg/mL [18]. Therefore, being higher in patients with early symptom onset than in later symptomatic phase, CSF p-NfH can be used as criteria for early inclusion of patients in clinical trials. While individually were stable over time in group comparison, CSF p-NfH were found to be significantly higher in patients with intermediate and fast disease progression, showing a weak prognostic value, but relevant in some subgroups of patients [18].

In the study led by Feneberg et al., CSF p-NfH discriminated patients with early symptoms from other neurological diseases and MND mimics, confirming their potential utility in precocious enrollment of patients in clinical trials. Moreover, they were higher in early versus later presenters [21].

## 4. Light Chain Neurofilament (NfL)

NfL is the lowest molecular weight subunit and the most abundant in axons. Given its high solubility and precocious diffusion from neural cells into CSF, it is rapidly detectable in biofluids after axonal degeneration. Accordingly, it presents high sensitivity in diagnosis of MND, being able to early identify ALS patients from DM. Nevertheless, NfL is an unspecific indicator of axonal damage, so its potential diagnostic role should be complemented with other neurological assessments [22].

Tortelli and colleagues found elevated CSF NfL levels in ALS respect to DC and identified a cut-off value of 1981 pg/mL discriminating between the two groups with a sensitivity of 78.4% and specificity of 72.5% [23]. In ALS patients CSF NfL concentrations showed a significant correlation with diagnostic delay, the ALSFRS-R and the progression rate, probably reflecting the burden of MNs degeneration.

Lu et al. measured NfL concentrations in serum, plasma and CSF in two cohorts of patients, and found higher levels in all biofluids in ALS compared to controls, discriminating with high sensitivity and specificity between ALS patients and HC [10]. Blood NfL levels at baseline were significantly higher in ALS-fast progressors than in ALS-slow progressors and correlate with progression rate at baseline (PRB) and progression rate at last visit (PRL). They detected also higher plasma NfL concentrations in female patients. In longitudinal measurement, there was a steady blood NfL expression over time. As a matter of fact, blood NfL levels at recruitment predicted survival independently from other clinical variables, while resulted to be stable over time in longitudinal samples, suggesting a role for NfL as a prognostic and pharmacodynamic biomarker.

CSF NfL concentrations were compared to measures of Diffusion Tensor (DT) techniques on brain Magnetic Resonance Imaging (MRI) by Menke and colleagues [11]. Higher CSF NfL levels were associated with reduced fractional anisotropy (FA) and increased radial diffusivity (RD) in corticospinal tracts (CSTs) in ALS patients compared to controls, suggesting a combined role of neurochemical and neuroimaging-based findings in assessing neurodegeneration in ALS.

Verde et al. identified a cut-off level of 62 pg/mL discriminating between ALS and DC, and between ALS and other neurological diseases, including neurodegenerative diseases, with high sensitivity and a relatively high specificity [24]. ALS patients presented higher serum NfL levels respect to controls, except for Creutzfeldt Jacob disease (CJD). In ALS group, serum NfL did not present a statistical relationship with site of onset, ALSFRS-R at sampling, treatment with riluzole, pathological state assessed with DTI and presence of *C9Orf72* or *SOD1* mutations.

Conversely, NfL showed a strongly significant correlation with progression rate and survival and, as demonstrated by other studies, they remained stable over time in longitudinal analysis [24]. In consideration of the early rise of NfL levels, followed by a stabilization during the remaining stages of the disease, the authors proposed the employment of this biological marker in the diagnostic process, for example in at-risk populations as a screening test or in those patients with recent onset of symptoms and not fulfilling El Escorial diagnostic criteria.

This is in agreement with the findings of Benatar and colleagues, who found increased serum and CSF NfL levels not only in ALS patients, but also in pre-symptomatic individuals as far back as 11.6 months before the onset of clinical signs or symptoms of the disease [12]. While longitudinally measurements of serum NfL were stable both in controls and in ALS patients, in at-risk individuals there was an increase over time of NfL levels, until at least 6 months after phenoconversion. No difference was found according to genetic mutations.

Nfs can be considered as markers for the extent of MND, indeed CSF p-NfH and NfL were reported to be increased with number of regions with both UMN and LMN involvement [18].

Confirming this hypothesis, Gaiani et al. correlated CSF NfL with subtypes of MNs disorders, identifying higher NfL levels in patients with atypical ALS, progressive bulbar palsy and UMN-dominant, in comparison with those with progressive muscular atrophy (PMA) and flail arm or leg syndrome [25]. Since MNs composing the CSTs are enriched with Nfs, the high amount of NfL detected in biological fluids would reflect damage and successive degeneration of pyramidal tracts. As shown by other studies, NfL levels were found to be constant over time, and patients with a more aggressive disease presented higher NfL concentrations. In this scenario, the measurement of NfL in patients with predominant LMN signs, would assume a prognostic value. Moreover, other than ALS, also FTD affected patients showed higher amount of CSF NfL.

Serum NfL were confirmed to correlate with UMN degeneration by Gille and collegues, and were reported to be independent predictors of survival in ALS patients [26]. Their levels were higher in ALS as compared to HC and hSP, probably due to a slow process of neurodegeneration, but not as compared to acute and chronic demyelinating polyradiculoneuropathy, denoting a low specificity of NfL for ALS.

In the prospective study of Steinacker et al. CSF NfL showed a high positive predictive value (PPV) in discriminating MND from other diseases, with a cut-off of 2200 pg/mL; besides they correlated with disease duration and progression [20].

CSF and serum NfL were found to be increased in patients with early symptoms from other neurological diseases and MND mimics; nevertheless, no difference in CSF and serum NfL concentrations have been found between early and later symptomatic phase, neither between fast and slow progressors [21].

Similarly to p-NfH, CSF and also blood NfL were reported to be massively elevated in symptomatic mutation carriers, while their levels are low and comparable in controls and asymptomatic carriers [13]. Moreover, Nfs levels were correlated with disease duration but not with severity. These findings confirmed Nfs as biomarkers of neurodegeneration.

In the study of Rossi and colleagues, CSF pNF-H and NfL levels were found to be increased in ALS with respect to patients with non-inflammatory neurological diseases and with acute/subacute inflammatory diseases and tumors, reaching the level of statistical significance only in the first group [27]. These findings suggest that reliability of Nfs as diagnostic biomarkers is limited when comparing ALS patients to diseases with elevated acute/subacute neuronal and axonal damage. No correlation was found between CSF Nfs and site of onset, age at onset and sex, while a weak, but significant, inverse correlation was found with diagnostic delay [27].

## 5. Discussion and Future Perspectives

Growing evidence have confirmed the potential utility of p-NfH and NfL as biomarkers in ALS, both in assessing diagnosis and predicting prognosis, and in monitoring treatment efficacy.

Considering its high sensitivity and its precocious elevation in biological fluids, NfL are promising diagnostic biomarkers in neurodegenerative disorders. They are able to discriminate accurately not only between ALS and HC, but also between ALS and DM; nevertheless, they lack specificity for MNs damage, since their levels showed relevant increase also in other neurodegenerative disorders. Therefore, serum and CSF NfL represent useful tools in diagnosis, especially in patients with high clinical suspicion for MND and when associated with other complementary assessments (clinical, radiological and electrophysiological biomarkers).

Conversely, the diagnostic utility of p-NfH has been extensively supported by the results of the above-mentioned studies. In particular, CSF p-NfH showed the higher diagnostic accuracy in discriminating ALS from both DM and DC in observational and retrospective studies [7,8,18,19,20].

A common matter in MND diagnosis is that a huge number of patients will not reach the requested criteria for definitive diagnosis in life or will reach them only in the ending stage of the disease: this issue could be overcome by adding some biological biomarkers to El Escorial Revised diagnostic criteria [28], and Nfs dosage in biofluids may represent a valid candidate.

Moreover, it must be taken into considerations the neuroanatomical correlate of Nfs elevation in biofluids: both p-NfH and NfL correlate with the extent of UMN and LMN involvement, although the first is primarily associated with LMN damage [18], while the latter better correlates with UMN and CSTs involvement [11], as demonstrated by DTI-MRI studies.

The vast majority of these works also assessed the prognostic value of blood Nfs in ALS.

Serum and plasma p-NfH concentrations were reported to peak in the early phase of the disease, especially in patients with shorted diagnostic delay and faster disease progression, probably reflecting the neurodegenerative process and the trend of neurological decline, and to fall in the later stages of the disease, when the pool of MNs has been already depleted. The trend of plasma p-NfH identified by Lu et al. and McCombe et al. [15,17] may reflect the kinetics of neurofilaments clearance from axons, which in turns depends on the rate of MNs degeneration in brain and spinal cord of ALS affected patients. Indeed, while during earlier stages of the disease the neurodegenerative process spreads from a region to another one, involving a great number of axons, and so provoking a rise in concentrations of Nfs detectable in blood and CSF, in later stages there is a decline in Nfs levels, given the few numbers of MNs left. In line with this, patients with slower disease progression presented stable p-NfH levels over time, reproducing a milder rate of neurodegeneration. For this reason, the measurement of p-NfH in cross-sectional studies has limited utility, since does not take into account disease heterogeneity in terms of diagnostic delay, disease progression and burden of MNs involvement. On the contrary, longitudinal studies allow one to follow p-NfH rise over time, enabling to predict disease severity when tested near symptoms onset, and to estimate disease progression by monitoring the change of p-NfH levels in the time. Similarly, several studies have shown steady expression of blood NfL in longitudinal measurements, making blood NfL a promising pharmacodynamic biomarker, useful to evaluate treatment response. Besides, serum and CSF NfL have also shown a strong prognostic relevance, since their concentrations at symptom onset predicted disease course. Furthermore, NfL levels are reported to predict phenoconversion in pre-symptomatic individuals.

Additionally, from a therapeutic perspective, the use of a non-invasive and relatively stable biomarker may be of paramount importance in potential clinical trials based on neuroprotective drugs. While in other diseases, like multiple sclerosis, CSF and blood Nfs have been tested in evaluating the effects of disease-modifying therapies, so far there are no studies investigating the use of Nfs as indicators of treatment response in ALS. This application would be very important in the current epoch, as new molecular therapies (i.e. antisense oligonucleotides in SOD1 and C9Orf72 mutated ALS) are coming to light in the therapeutic scenario of MND.

In conclusion, the diagnostic clinical relevance of Nfs dosage in biofluids, along with the positive correlation with disease progression and the easy accessibility of this biomarkers, points towards the standardization and validation of Nfs measurements in clinical activity. Nonetheless, they could be employed for improving early diagnosis and monitoring treatment efficacy, thus representing promising biomarkers in observational and interventional clinical trials.

## Figures and Tables

**Table 1:** Neurofilament studies in amyotrophic lateral sclerosis. The most relevant studies about neurofilaments (Nfs) in Amyotrophic lateral sclerosis (ALS). For each study, we reported the first author, the study design, the subtype of Nf analyzed (phosphorylated heavy chain and light chain Nfs, or both), the biological fluid analyzed (plasma, serum or cerebrospinal fluid), the features of the participants, the main findings of the study, the diagnostic and prognostic relevance of the studies, and when available, the cut-off level of Nfs able to discriminate patients from controls.

Authors	Study Design	Neurofilaments	Tissue Analysed	Participants	Main Findings	Diagnostic and/or Prognostic Relevance	Cut-Off
Boylan et al. [2]	Longitudinal; pilot (cross-sectional)	p-NfH	Plasma, serum, CSF	43 ALS affected patients (20 patients longitudinally followed)	Higher p-NfH levels were associated with faster decline in ALSFRS-R and shorter survival. Plasma p-NfH were higher in patients with bulbar rather than spinal onset.	p-NfH could be reliable biomarkers of disease activity and progression in ALS.	
Lu et al. [3]	Cross-sectional; longitudinal prospective	p-NfH	Plasma	136 patients with ALS (74 of them longitudinally followed), 104 healthy and neurological controls	Fast progressing patients have higher p-NfH levels than controls at an early stage and lower levels at late disease stage.	Trajectories of plasma p-NfH reflect the speed of neurological decline. Cross-sectional measurement of plasma p-NfH have a limited prognostic role in ALS.	
McCombe et al. [4]	Longitudinal prospective	p-NfH	Serum	98 ALS patients and 61 healthy controls	p-NFH increased over time in early stage of disease, and the rate of rise inversely correlated with survival; slow-progressing patients have lower p-NfH concentrations.	Initial level of p-NfH is a marker of disease severity and changes over time are marker of disease progression.	
Li et al. [5]	Cross-sectional	p-NfH	Plasma and CSF	51 ALS patients, 12 MSA patients and 30 HC	CSF and plasma p-NfH were higher in patients than controls and correlated to each other. CSF more than plasma p-NfH correlated with time to generalization.	p-NfH and TTG could be indicators of disease survival.	685 pg/ml for p-NfH in plasma (sensitivity of 80.39% and specificity of 73.81%), and 589 pg/mL for CSF, with a sensitivity of 82.35% and a specificity of 73.81%
De Schaepdryver et al. [6]	Longitudinal retrospective	p-NfH	Serum and CSF	85 ALS patients, 215 DC and 31 ALS mimics	CSF and serum p-NfH were increased compared to DC and ALS mimics, and in ALS patients they correlated with disease progression. Serum p-NfH correlated inversely with symptom duration. CSF p-NfH correlated with burden of UMN and LMN involvement.	The diagnostic potential of p-NfH measurement in CSF, more than serum, could be used as a criterion of inclusion in clinical trials.	750 pg/mL for CSF p-NfH discriminating ALS from mimics, with an elevated sensitivity and specificity (92.9% and 96%, respectively). A cut-off of 81.9 pg/mL in serum distinguished ALS from mimics with lower sensitivity and specificity (7.8% and 85.2%, respectively).
Zucchi et al. [7]	Cross-sectional	p-NfH	Serum and CSF	30 patients, among who 14 with UMN-dominant ALS, 7 with PLS and 9 with hSP, and 9 HC	ALS patients have higher serum and CSF p-NfH concentrations compared to HC and hSP. CSF p-NfH predicted survival in ALS patients.	Role of CSF p-NfH as a prognostic biomarker in diseases presenting with UMN signs; serum and CSF p-NfH may have a diagnostic relevance.	
Gendron et al. [8]	Cross-sectional and longitudinal	p-NfH	CSF	135 C9Orf72 expansion carriers (asymptomatic, ALS/ALS-FTS or FTS) and 107 noncarriers (healthy, ALS/ALS-FTD or FTD); 37 carriers and 17 noncarriers were followed longitudinally.	CSF p-NfH discriminated symptomatic and asymptomatic carriers and predict disease severity and surival in C9-ALS. Higher p-NfH were associated with faster disease progression and shorter survival in C9-ALS.	Use of CSF p-NfH as a prognostic biomarker in clinical trials, especially in patients with hexanucleotide expansion in C9Orf72 gene.	176 pg/mL in CSF was able to discriminate with elevated sensitivity and specificity (98.8% and 96.4%, respectively) between symptomatic and asymptomatic *C9Orf72* mutation carriers.
Tortelli et al. [9]	Cross-sectional	NfL	CSF	37 ALS patients, 25 patients with CIDP and 21 patients affected by other neurodegenerative diseases.	CSF NfL were higher in ALS patients than controls and showed correlations with diagnostic delay, the ALSFRS-R and the progression rate, probably reflecting the burden of MNs degeneration.	NfL may be useful marker of disease activity and progression in ALS.	1981 pg/mL in CSF discriminated between ALS and DC with a sensitivity of 78.4% and specificity of 72.5%.
Lu et al. [10]	Longitudinal, observational (two cohorts)	NfL	Serum, plasma and CSF	103 ALS patients and 42 HC (cohort 1); 64 ALS patients and 36 HC (cohort 2)	Blood NfL levels at baseline were higher in fast and correlate with progression. In longitudinal measurements blood NfL were stable over time.	Blood NfL levels are strong predictors of survival, independently from other clinical variables. Given this stability over time, NfL may be reliable pharmacodynamic biomarkers.	CSF, serum, and plasma NfL discriminated patients with ALS from healthy controls with high sensitivity (97%, 89%, 90%, respectively) and specificity (95%, 75%, 71%, respectively).
Menke et al. [11]	Cross-sectional	NfL	Serum and CSF	25 ALS patients and 17 HC	ALS patients have higher NfL levels and CSF NfL concentrations correlated with clinical and imaging UMN burden, and with rate of disease progression.	Combined role of neurochemical and neuroimaging-based findings in assessing neurodegeneration in ALS.	
Verde et al. [12]	Longitudinal prospective	NfL	Serum	124 ALS patients, 50 patients without neurodegenerative diseases, 44 patients with disease mimics and 65 patients with other neurodegenerative diseases.	ALS patients presented higher serum NfL levels respect to controls, except for CJD. Serum NfL showed a strong correlation with progression rate and survival and they remained stable over time in longitudinal analysis.	The authors proposed the use of serum NfL as a diagnostic biomarker, useful in at-risk populations as a screening test or in patients with recent onset of symptoms and not fulfilling El Escorial diagnostic criteria.	A cut-off level of 62 pg/mL discriminated between ALS and disease mimics with a sensitivity of 85.5% and a specificity of 77.3%; a cut-off level of 49 pg/mL discriminated between ALS and non-neurodegenerative controls.
Benatar et al. [13]	Longitudinal prospective	NfL	Serum and CSF	84 individual at-risk of developing ALS, 17 ALS patients, 34 controls and 10 phenoconverters.	Serum and CSF NfL levels were higher in ALS patients and in pre-symptomatic individuals as far back as 11.6 months before the onset of ALS, than in controls and at-risk individuals.	Serum NfL provide a new tool to quantify pre-symptomatic disease progression and to potentially predict the time of phenoconversion.	
Gaiani et al. [14]	Longitudinal retrospective	NfL	CSF	94 ALS patients, 20 FTD patients, 18 patients with motor neuropathies and 44 controls.	Higher NfL levels were found in patients with atypical ALS, PBP and UMN-dominant, in comparison with PMA and flail arm or leg syndrome.	Low NfL levels in patients with predominant LMN signs may be prognostic indicator of milder phenotype of disease.	
Gille et al. [15]	Cross-sectional; longitudinal (16 ALS patients)	NfL	Serum	149 ALS patients (among whom 15 *C9Orf72* mutation carriers, 15 with ALS-FTD), 19 ALS-mimics.	Serum NfL levels were higher in ALS as compared to HC controls and hSP, but not as compared to GBS and CIDP.	Serum NfL were independent predictors of survival in ALS but have low specificity as diagnostic biomarker.	
Steinacker et al. [16]	Longitudinal prospective	p-NfH and NfL	CSF	253 patients with MND (among whom 242 with ALS, 11 with PLS and 20 fALS), 85 with MND mimics, 28 with AD, 26 with Parkinsonian syndromes, 33 with polyneuropathies and 30 with facial palsies.	CSF Nfs levels were increased in MND as compared to MND mimics and HC. Nfs levels were associated with MND progression and disease duration.	CSF Nfs have a high relevance in the differential diagnosis of MNDs.	They found a cut-off level of 2200 pg/mL for NfL, with sensitivity of 77%, a specificity of 85% and a PPV of 87%. For pNfH, a cut-off of 560 pg/mL with 83% sensitivity, 77% specificity and 82% PPV was obtained.
Weydt et al. [17]	Cross-sectional	p-NfH and NfL	Serum and CSF	12 asymptomatic and 64 symptomatic ALS mutations carriers and 19 family controls.	CSF p-NfH and serum and CSF NfL increased at early symptom onset in symptomatic carriers.	Blood and CSF Nfs are markers of structural axonal damage.	
Poesen et al. [18]	Cross-sectional; longitudinal (17 patients)	p-NfH and NfL	CSF	220 patients with ALS, 316 DC and 50 DM	CSF Nfs were lower in slower disease progressors and were correlated to the number of regions with both UMN and LMN involvement.	CSF p-NfH are specific for MND, have a diagnostic relevance in ALS and can be used as criteria for early inclusion of patients in clinical trials.	pNfH discriminated ALS patients from DM with a sensitivity of 90.7%, a specificity of 88.0% and a likelihood ratio of 7.6 at a cutoff of 768 pg/mL.
Feneberg et al. [19]	Cross-sectional multicenter	p-NfH and NfL	Serum and CSF	Patients with ALS at <6 months from symptom onset (54 CSF and 45 serum) or at >6 months from symptom onset (135 CSF and 118 serum), patients with other neurological disease (65 CSF and 48 serum), patients with MND mimics (27 CSF and 21 serum) and patients with other MND (21 CSF and 16 serum).	CSF and serum NfL and CSF p-NfH were higher in patients with ALS than in controls. No difference was found between early and later symptomatic phase, neither between fast and slow progressors.	Serum and CSF Nfs effectively discriminate early ALS patients from neurological controls.	
Rossi et al. [20]	Cross-sectional	p-NfH and NfL	CSF	190 ALS patients (of whom 10 with ALS-FTD) and 130 controls, divided into patients with non-inflammatory neurological diseases and patients with acute/subacute inflammatory neurological diseases and tumors.	ALS patients have higher CSF Nfs as compared to patients with non-inflammatory neurological diseases. There was a weak inverse correlation with diagnostic delay.	The reliability of Nfs as diagnostic biomarkers is limited when comparing ALS patients to diseases with elevated acute/subacute neuronal and axonal damage.	

AD: Alzheimer’s disease, ALS: amyotrophic lateral sclerosis, ALSFRS-R: ALS Functioning Rating Scale Revised, CIDP: chronic inflammatory demyelinating polyneuropathy, CJD: Creutzfeldt Jacob disease, CSF: cerebrospinal fluid, DC: disease controls, DM: disease mimics, fALS: familial ALS, FTD: frontotemporal dementia, GBS: Guillan-Barré syndrome, HC: healthy controls, hSP: hereditary spastic paraplegia, LMN: lower motor neuron, MND: motor neuron disease, MNs: motor neurons, MSA: Multiple System Atrophy, NfL: neurofilament light chain, Nfs: neurofilaments, PBP: progressive bulbar palsy, PLS: primary lateral sclerosis, PMA: progressive muscular atrophy, p-NfH: phosphorylated neurofilament heavy chain, PPV: positive predictive value, TTG: time to generalization, UMN: upper motor neuron.
